# Preparation and characterization of holmium doped ZIF-8 nanocrystals for white light emitting phosphors

**DOI:** 10.1038/s41598-025-33503-8

**Published:** 2026-01-30

**Authors:** Mariam Sh. Gohr, Hanan Ali, Sara Gad, Hesham M.A. Soliman, Alaa E. Giba

**Affiliations:** 1 Composites and Nanostructured Materials Research Department, Advanced Technology and New Materials Research Institute (ATNMRI), City of Scientific Research and Technological Applications (SRTA-City), New Borg Al-Arab City, 21934 Alexandria Egypt; 2 National Institute of Laser Enhanced Sciences, Cairo University, Giza, 12613 Egypt; 3 Electronic Materials Research Department, Advanced Technology and New Materials Research Institute (ATNMRI), City of Scientific Research and Technological Applications (SRTA-City), New Borg Al-Arab City, 21934 Alexandria Egypt

**Keywords:** Metal-organic framework, ZIF-8, Lanthanides, Holmium, White light emission, Luminescent stability., Chemistry, Materials science, Nanoscience and technology, Optics and photonics

## Abstract

We report on the generation of white light from Ho-doped ZIF-8 nanocrystals synthesized by a simple wet chemical technique at room temperature. The influence of Holmium (Ho) concentration and heat treatment on both structural and optical responses has been investigated. The crystal structure and morphology of as-synthesized (As) and post-thermal annealed (TA) nanocrystals have been examined using X-ray diffraction (XRD) and Transmission Electron Microscope (TEM).  In addition, the thermal stability and bond structure have been studied via Thermogravimetric analysis (TGA) and Fourier Transform Infrared Spectroscopy (FTIR), respectively. Moreover, the photoluminescence (PL) measurements were performed using two excitation wavelengths: 445 nm and 355 nm. The XRD and TEM evidenced the sodalite nanocrystalline nature of the samples. The TGA and FTIR analyses demonstrated the degradation behavior of ZIF-8 due to the incorporation of Ho ions and thermal calcination. The PL results showed intense luminescence response, in particular, from the heat-treated samples. Furthermore, the PL exhibited a naked-eye observed white light emission. This was attributed to the structural modifications induced by the dopant and thermal treatment. Our findings could be used as a guideline towards the potential application of lanthanide-doped MOFs (ZIF-8) in light-emitting purposes.

## Introduction

Metal-organic frameworks (MOFs) are multipurpose materials with numerous applications in various fields, such as catalysis, optoelectronics, environmental remediation, energy, and biomedicine^1, 2^. MOFs are coordination polymers with high porosity and surface area^3^. It combines metal clusters and organic ligands to create a diverse range of structures^4^.Among them, Zeolitic imidazolate framework-8 (ZIF-8) is a type of MOF with a zeolite skeleton structure. It has an adaptable structure, high porosity, in addition to high thermal stability, good chemical stability, and is easily synthesized^5, 6, 7^. It is found that the cage structure of ZIF-8 crystals, in particular, may efficiently isolate the fluorescent groups enclosed in it, while inhibiting aggregation that induces fluorescence quenching^8, 9^. Moreover, by improving the dispersion of ZIF-8 particles, the shape and size of ZIF-8 crystals can be highly uniform^10^. Based on this, ZIF-8 crystals were effectively self-assembled into colloidal crystals with superstructures^11^. This colloidal ZIF-8 crystal provides an effective technique to realize high-performance white light emitting diodes (WLEDs)^8^.Furthermore, ZIF-8’sporous structure allows for sufficient area to contain luminescent guest molecules^12^. These hosted molecules can enhance, modify, or add new features to ZIFs, allowing properties to be tailored to desired objectives. In this regard, lanthanide (Ln)-coordinated metal-organic frameworks (Ln-MOFs) are of great interest due to the peculiar emissions of the Ln^3+^ ions that go beyond the intrinsic emission produced through luminous guest molecules (intercalated inside MOFs) and organic links. Because of their superior optical qualities, Ln-MOFs are preferred and used in a variety of applications, including photo-catalysis, optical thermometry, sensing, storage, and light-emitting diodes (LEDs)^13, 14, 15^. The Guest-MOF framework structure has been exploited by researchers targeting white light-emitting applications. Xiang et al.^16^have succeeded in preparing Fluo@ZIF-8 and RhB@ZIF-8 thin films that were mixed with a 1:1 mass ratio, resulting in green-to-red emission. Those films were paired with a blue LED chip to produce white light emission^16^. Also, yellow-green-emitting Alq3 encapsulated in blue-emitting Zn-MOF has been employed to create white light-emitting diodes (WLEDs)^17^. In addition, CsGeBr_3_ coupled with Eu-MOF was recommended for WLED devices^18^. Moreover, Eu and Dy ions have also been doped in oxide-based phosphors for lightning applications^19, 20^. Furthermore, co-doped Er with Yb and/or Eu ions into MOFs has been reported as a dual-mode light emitting phosphor for potential WLED^21^. As a result, blue, green, and red emission lines from that phosphor have been obtained. In the same direction, Tb, Dy, and Eu tri-doped MOFs have also been used for generating the three colour components of white light^22^. However, doping with several Ln^3+^ ions would increase the structural complexity. Thus, achieving white light emission using a smaller number of doped Ln^3+^ is valuable. Despite the study of different lanthanide ions in MOFs, single-doping of Ho in ZIF-8 has not been reported elsewhere. The significance of Ho ions appears in their remarkable green emission response^23– 25^. This makes Ho a favourable green emitter in luminescent devices such as WLED^26, 27^. In addition, the electronic structure of Ho ions owns a specific lasing transition in the infrared region (≈ 2 μm), which is exploited in Ho-based solid-state lasers.^28, 29^ Also, the magnetic characteristics alongside the bio-compatibility of Ho triggers related applications in bio-imaging^30^. Thus, among the other reported Ln^3+^ (e.g. Eu, Tb, Dy, or Er) doped in MOFs, Ho exhibits interesting luminescence responses either in the visible or IR region as well as magnetic and bio-compatibility characterizations. Owing to the above diverse applications and some missing reported information, the present study is motivated to fill the scientific gap of this couple, Ho-doped ZIF-8. Herein, a simple one-pot synthesis process prepared at room temperature for Ho-doped ZIF-8 MOFs nanocomposites is presented . The influence of Holmium dopant concentration as well as the heat treatment on the ZIF-8’s crystal structure, thermal, and luminescence properties is investigated. As a result, these nanocrystals show great potential as a new material for future WLED applications.

## Experimental section

### Materials

Zinc (II) nitrate hexahydrate (Zn(NO_3_)_2_.6H_2_O, 99%), methanol (CH_3_OH, 99%HPLC analytical grade), 2-methylimidazole (C_4_H_6_N_2_, 99%), and Holmium nitrate (III) pentahydrate (Ho(NO_3_)_3_·5H_2_O, 99%) were purchased from Sigma-Aldrich. The above-mentioned chemicals possess analytical purity and were used without any purification. The entire experiment was performed using deionized water.

### Synthesis of the undoped ZIF-8 nanocrystals

ZIF-8 nanocrystals were synthesized according to the previous method^14^. Specifically, 0.740 g (2.5 mmol) of Zn (NO_3_)_2_.6H_2_O in (MeOH) (50 ml)was added to 50 ml of MeOH containing 1.620 g (20 mmol) of 2-Mim and stirred for 24 h at room temperature (25 ◦C)at neutral PH. The product was centrifuged (6000 rpm, 20 min), washed three times with MeOH, and then dried at 60 ◦C overnight.

### Synthesis of Ho-doped ZIF-8: x% Ho (x = 1, 3 or 5%) nanocomposites

For the synthesis of Ho-doped ZIF-8, a solution containing 2-Mim (1.620 g, 20.0 mmol), Zn(NO_3_)_2_⋅6H_2_O (0.740 mg, 2.5 mmol), and Ho(NO_3_)_3_⋅5H_2_O (22.1,55 and 110.3 mg, 0.05,0.15, and 0.25 mmol) was dissolved in 50, 40, and 10 mL MeOH, respectively. Afterwards, the above-mentioned solutions were mixed, followed by stirring for 24 h at room temperature (25 ◦C). The product was centrifuged (6000 rpm, 20 min), washed three times with MeOH, and then dried at 60 ◦C overnight. To investigate the effect of thermal treatment on the prepared nanocomposites, all powders were calcined at 350 ◦C for 2 h with a heating rate of 2 °C min^− 1^in ambient atmosphere. Throughout the whole text, the following samples notations are used: **ZIF-8 (% Ho)_As** refers to the as-synthesized ZIF-8 nanocrystals prepared at room temperature without any post-synthesis thermal treatment. **ZIF-8 (%Ho)_TA** denotes the thermally annealed ZIF-8 samples subjected to controlled heat treatment at 350 °C for 2 h.

### Characterization of undoped and doped ZIF-8 nanocrystals

Powder X-ray diffraction (XRD) data were obtained at room temperature using a Shimadzu X-ray diffractometer (model XRD-6000) running at 30 kV and 30 mA and equipped with a Ni filter and Cu Kα (λ = 1.5406 Å) radiation. Fourier transform infrared (FT-IR) spectroscopy was conducted using a Varian spectrometer (model 660-IR) fitted with a Pike single-bounce diamond/ZnSe ATR cell. High-resolution transmission electron microscopy (HRTEM) images were acquired on a low-vacuum transmission electron microscope (TESCAN, model MIRA3), accompanied by EDX spectrometry for analyzing chemical components found in synthesized composites. Photoelectron spectroscopy (XPS) was performed using K-Alpha X-ray photoelectron spectrometer, Thermo Fisher Scientific-US. The thermogravimetric analysis (TGA) was accomplished by using a TGA Instruments thermal analyzer (model SDT 640) in an N_2_ atmosphere with a flow rate of 50 mL min^− 1^. The nitrogen adsorption and desorption isotherms at 77 K were measured with a Quantachrome NOVA porosimeter (model 2200E). Before measurements, the samples were vacuum-degassed for 12 h at 150 °C (+ 80).The PL measurements were performed using a 445 nm cw-diode laser for the visible excitation approach. In UV excitation, the 3rd harmonic 355 nm line of a YAG: Nd laser with 40 ns, 70 mJ, and 10 Hz pulse duration, pulse energy, and repetition rate, respectively, was used. The emission from the samples was collected on an optical fiber and detected by a compact spectrometer (Thorlabs-CCS100).

## Results and discussion

**Fig. 1:**
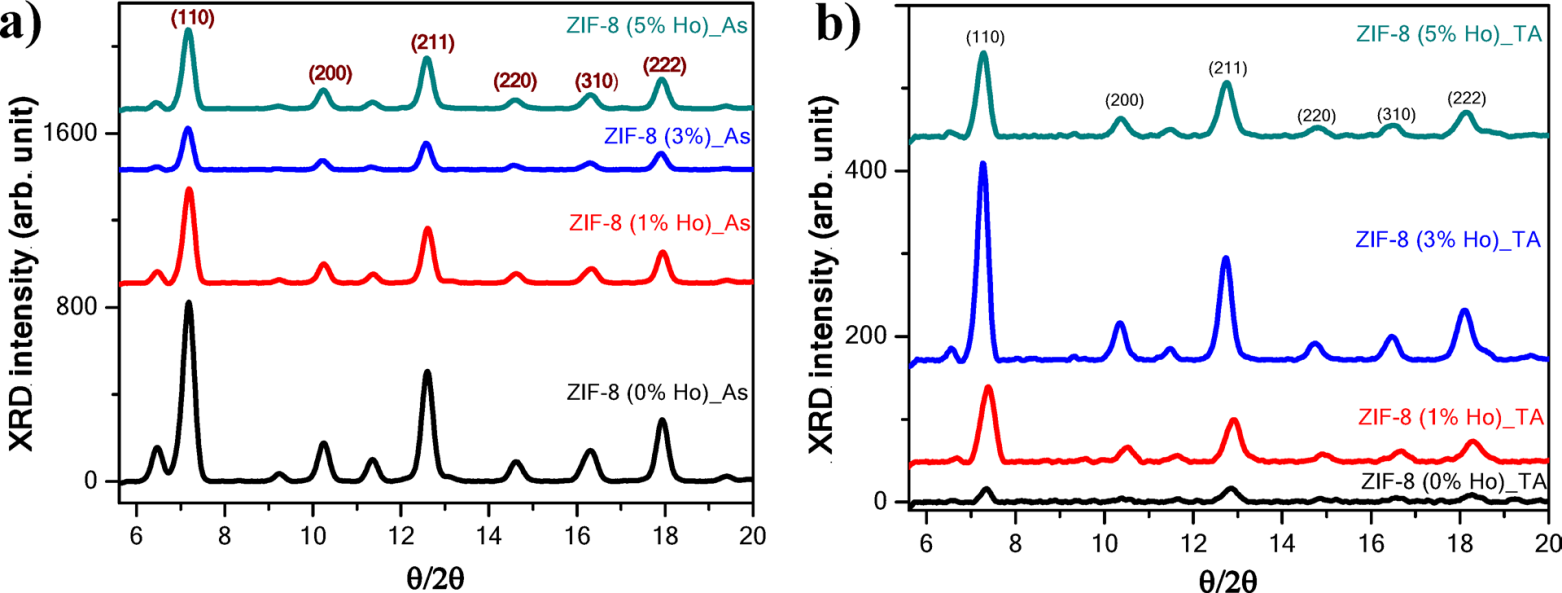
XRD patterns of undopedZIF-8 and Ho-doped ZIF-8 for (**a**) as-synthesized (As) and (**b**) thermal annealed (TA) nanocomposites.

XRD of the as-synthesized (As) and post-thermal annealing (TA) samples has been performed to investigate the influence of Ho doping and thermal treatment on the formed phase structure. Figure (1a) illustrates the diffractograms from the as-synthesized nanocrystals at different Ho concentrations. All patterns confirm the crystalline features of theZIF-8 framework structure in line with previous reports^31, 32^. It confirms that the fundamental crystal structure of ZIF-8 remains largely intact without major structural transformation owing to the incorporation of Ho ions. The pattern of ZIF-8 (0% Ho)_As shows diffraction peaks located at 2θ of 7.5^◦^, 10.5^◦^, 12.6^◦^, 14.8^◦^, 16.5^◦^, and 18.1^◦ ^which correspond to the planes (110), (200), (211), (220), (310), and (222), respectively^32^.The dominant XRD peak is related to the (110) plane as a preferred orientation. It can be noticed that the doping of Ho induces degradation in the XRD intensity. The ZIF-8 (3% Ho)_As sample exhibits the lowest XRD intensity. This degradation infers a short-order range of crystals induced by the dopant. It can be suggested that the MOF framework structure experiences a deformation due to the guest atoms/molecules. Although the change appears in the XRD intensity, due to doping, could indicate a change in the crystallite size, the full width at half maximum (FWHM) of the preferred orientation at (110) looks similar. In this regard, the average crystallites size and microstrains of nanocrystals can be calculated according to the Williamson-Hall (W-H) method. Equation (1) represents the W-H formula, where β is the full width at half maximum (FWHM), D is the crystallites size (nm), and ε is the microstrain.

1 $$\beta {\text{ }}cos\theta {\text{ }} = {\text{ }}4{\text{ }}\varepsilon {\text{ }}sin\theta {\text{ }} + {\text{ }}0.9{\text{ }}\lambda {\text{ }} / {\text{ }}D$$

The crystallites size and microstrain of nanocrystals with different Ho concentrations have been estimated and stated in Table 1.It can be observed that Ho-doping induces an increase in crystallite size. On the other hand, the XRD of nanocrystals after the heat treatment, as shown in Fig. 1b, reveals a significant degradation in their XRD peak intensity compared to the as-synthesized counterpart; however, the ZIF-8 (3% Ho)_TA sample exhibits less XRD intensity degradation.

**Table 1:** The crystallites size (nm), dislocation density, and microstrains of undoped ZIF-8 and Ho^3+ ^doped ZIF-8 with different Ho^3+^ mole ratios for (a) as-synthesized and (b) thermal annealed nanocomposites.

	a) As-Synthesized (As)				b) Thermal Annealed (TA)			
Sample code	Crystal Size (D) (nm)	Dislocation Density (δ) (×10⁻³ nm⁻²)	Microstrain (ε)	Structure	Crystal Size (D) (nm)	Dislocation Density (δ) (×10⁻³ nm⁻²)	Microstrain (ε)	Structure
ZIF-8 (0% Ho)	83.39	1.438	0.65	Hexagonal	23.582	0.0018	0.041	Hexagonal
ZIF-8 (1% Ho)	85.65	1.363	0.37	Hexagonal	35.08	8.13e^− 4^	0.026	Hexagonal
ZIF-8 (3% Ho)	88.04	1.290	0.031	Hexagonal	43.48	5.28e^− 4^	0.023	Hexagonal
ZIF-8 (5% Ho)	86.62	1.333	0.012	Hexagonal	73.86	0.29464	0.0572	Hexagonal

**Fig. 2:**
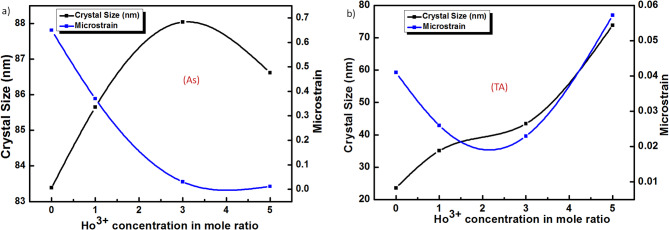
The relation between the crystallite size D (nm) and Microstrain vs. Ho^3+^ concentration in mole ratios for (**a**) as-synthesized (As) and (**b**) thermal annealed (TA) nanocomposites.

**Fig. 3:**
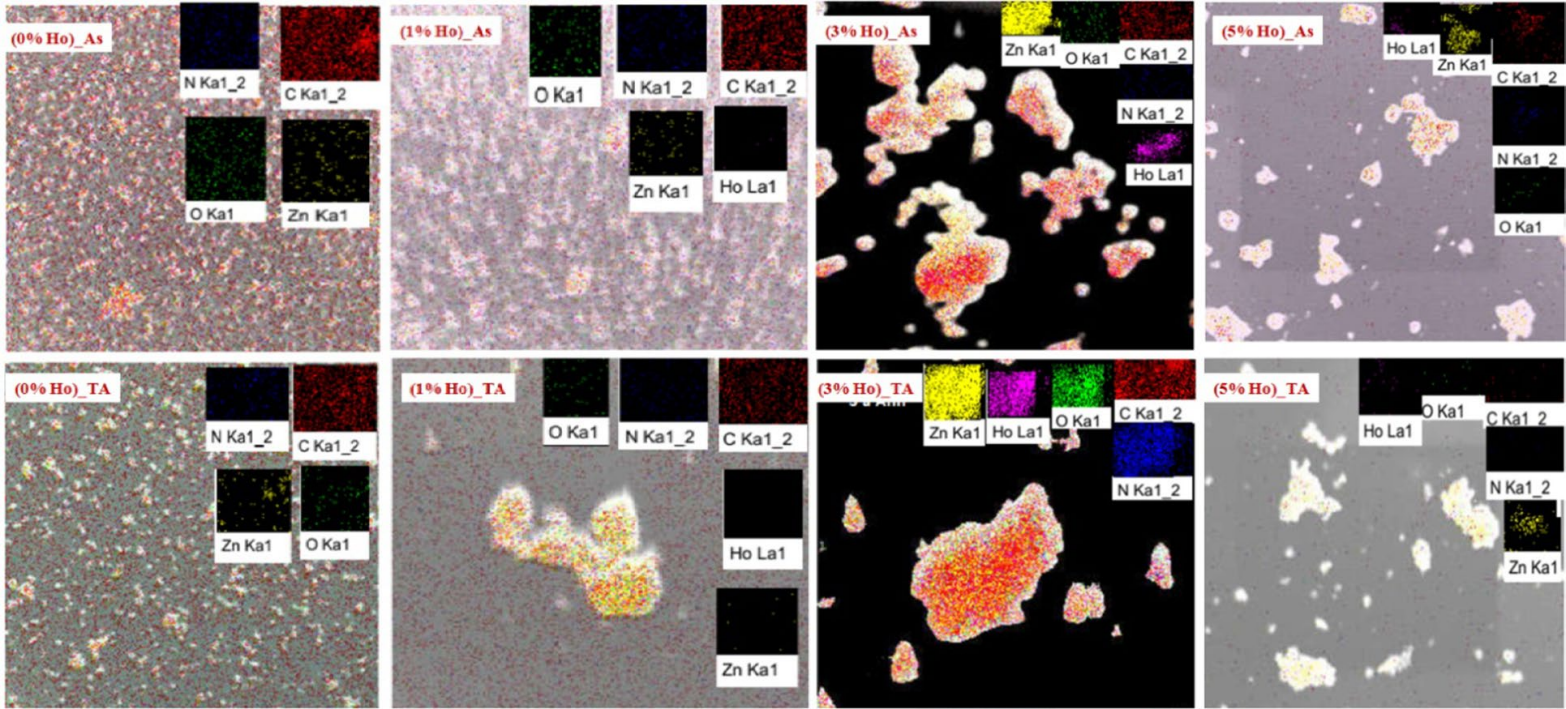
EDS mapping images of undoped ZIF-8 and Ho-doped ZIF-8 for as-synthesized (Top panel) and thermal annealed (Bottom panel) nanocomposites.

This implies the structural sustainability of this sample against the heating process compared to the others. The observed reduction in dislocation density and lattice microstrain infers fewer crystallographic defects, suggesting that Ho-doping alongside annealing helps to relax the crystal lattice (see Fig. 2).The microstrain values exhibit a downward trend, indicating lower internal lattice distortion with increasing dopant concentration. Even though ZIF-8 (3% Ho)_As sample, of the lowest XRD intensity, exhibits the largest crystallites size, accompanied by the lowest dislocation density value and considerably low lattice microstrain in both behaviors; before and after thermal treatment. This indicates a remarkable feature of this sample, ZIF-8 (3% Ho). For the compositional and morphological analyses, EDS mapping is examined. The EDS mapping images represent the morphological behavior of the samples at different concentrations of Ho ions before and after annealing, as well as exhibit the total distribution of the elemental compositions, see Fig. 3. It can be observed that the presence of Ho induces structural aggregations, which are further manifested after thermal treatment. However, all the elemental species have been found in a homogeneous distribution within the investigated area. The quantitative analyses almost agreed on the nominal average amount of Ho increasing from 1% to 5% with ± 0.5%. In addition, the amounts of C, N, O, and Zn have been varied from one sample to another, with average weights of 80%, 10%, 3%, and 7%, respectively.

**Fig. 4:**
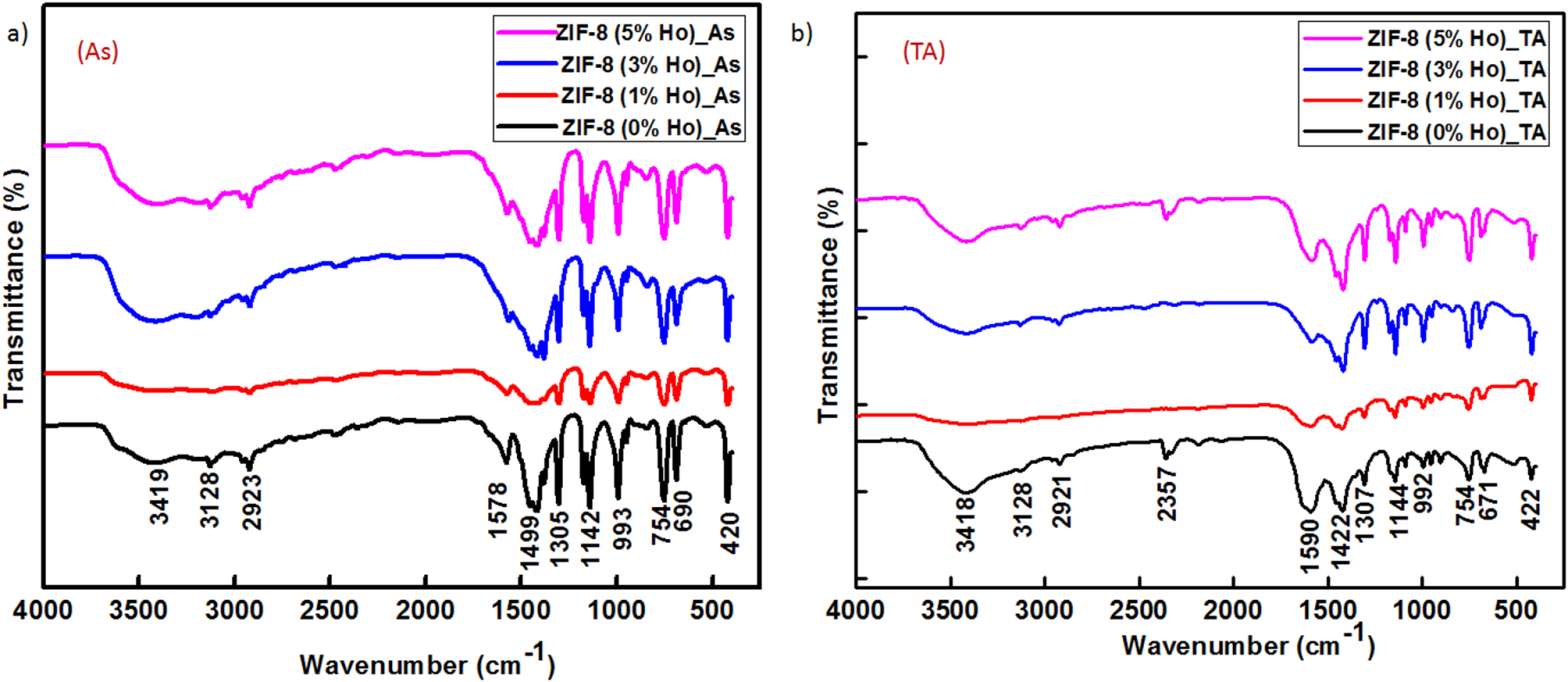
FTIR spectra of undopedZIF-8 and Ho-doped ZIF-8 for (**a**) as-synthesized (As) and (**b**) thermal annealed (TA) nanocomposites.

For gaining more information on the structure bonding, FTIR measurement has been performed as shown in Fig. 4. FTIR provides more detailed information on the chemical bond network of the synthesized nanocomposites before and after heat treatment. Guided by the previous literature, the obtained FTIR spectra of ZIF-8 can be assigned as follows: The band at 3419 cm^− 1^ corresponds to N–H elongation vibration and O–H stretching of adsorbed water molecules in the MOF pores. The observed peaks at 3128 and 2923 cm^− 1^ are attributed to asymmetric C–H stretching related to the carbons sp^2^ and sp^3^, respectively, existing in the structure of the imidazole ligand. Besides, the peaks appeared at 1587 and 1499 cm^− 1^ are attributed to the C–C and C = N stretching. Furthermore, the peaks at 1305 to 1499 cm^− 1^ are related to the stretching of the ring, and a wide band at 1142 cm^− 1^ is due to the aromatic C–N stretching. Vibration peaks appear at 993 and 754 cm^− 1^correspond to C–N stretching and C–H bending modes, respectively. In addition, the characteristic Zn–N stretching vibration was detected at 420 cm^− 1^, indicating a chemical combination of nitrogen atoms and zinc ions and the formation of ZIF-8[31-33]. In the case of doping ZIF-8 with Holmium before annealing, the peaks appear at 1578–1142 cm^− 1^with a slightly decreased strength, indicating that the MOF’s chemical structure changed, probably as a result of a small amount of formed Ho–N bonds. Furthermore, the samples underwent a heat treatment at 350 °C for 2 h, and FTIR spectra were also recorded and shown in Fig. 4b.

**Fig. 5:**
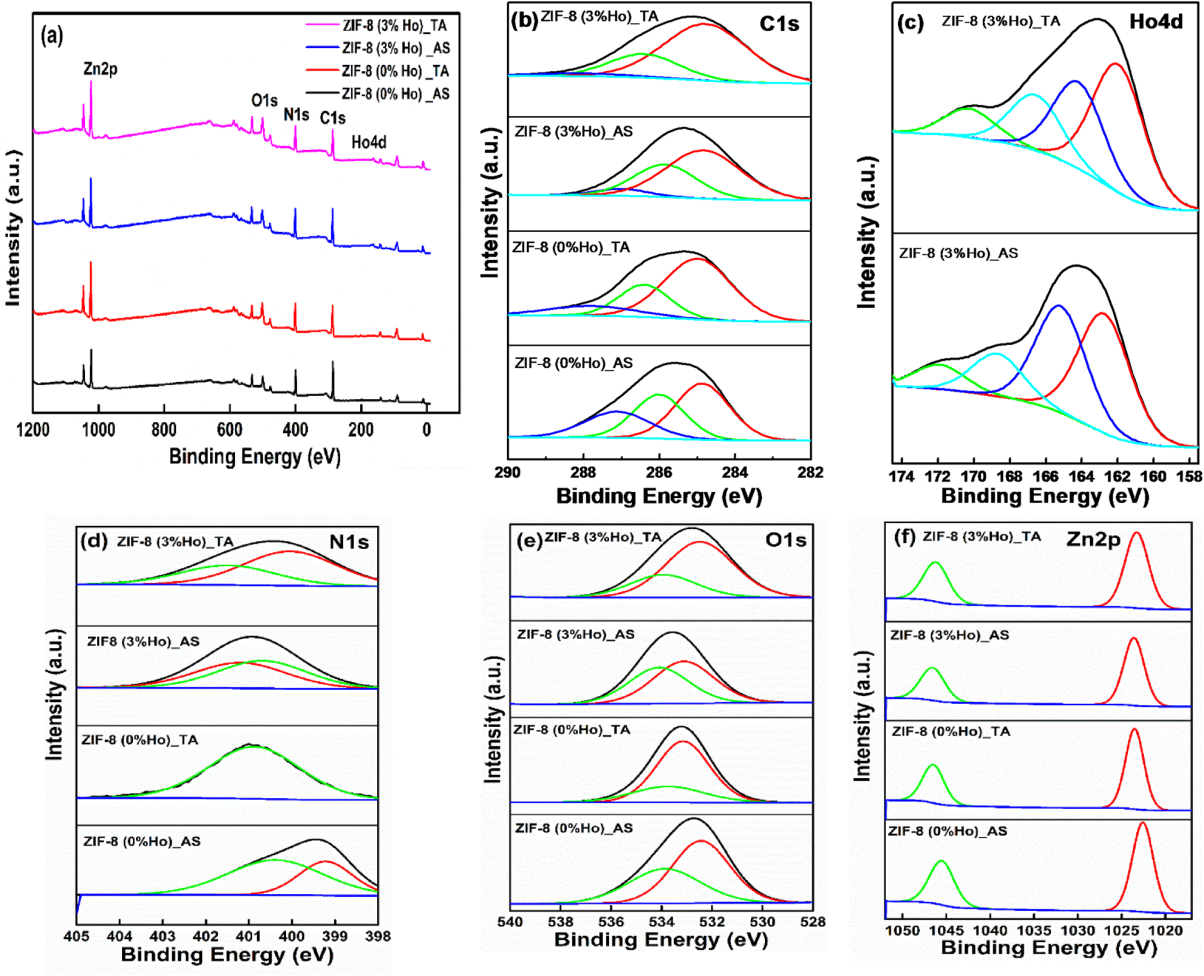
XPS spectra (**a**) wide survey, (**b**) C1s, (**c**) Ho3d, (**d**) N1s, (**e**) O1s, and (**f**) Zn2p of ZIF-8 (0%Ho)_AS, ZIF-8 (0%Ho)_TA, ZIF-8 (3%Ho)_AS, and ZIF-8 (3%Ho)_TA, respectively.

The synthesized nanocomposites retain their ZIF-8 structure even after heat treatment, although there is a noticeable decrease in the broad absorption bands (which is caused by hydrogen bonds formed between Zn metal and imidazole molecules) at wavenumbers in the 2923–3420 cm^–1^ range. This indicates that undoped and Ho-doped ZIF-8 still have their network character^33^.As a result, higher temperatures and/or longer annealing times for ZIF-8 would be necessary for a full breaking of the Zn-ligand linkages^32, 34^. These results are in agreement with XRD characterizations that show the conservation of the main features of the ZIF crystal structure. In this line, photoelectron spectroscopy (XPS) measurement would give more extensive information about the chemical compositions and states of the composite. The XPS has been performed to investigate the elemental composition and chemical states of the synthesized samples. Figures (5a) shows the survey spectra of the ZIF-8 (0%Ho) and ZIF-8 (3%Ho) samples before (As) and after annealing (TA). The signals related to the elements of Zn 2p, N 1 s, O 1 s, and C 1 s, with an additional signal assigned to Ho 4 d in doped samples can be detected. This confirms the composition of ZIF-8 and the incorporation of Ho ions in the composite. For better analyzing the XPS signals for each element, separate panels of deconvoluted spectra are involved in Fig. 5 (b-f) for different binding energy ranges. In the **C 1s** element (Fig. 5b), the deconvoluted peaks are located at binding energies around 284.7, 286.8, and 287.5 eV, which are assigned to graphitic carbon/C-sp^3^, C = C/C-O, and C = N/C = O bonds, respectively. The overall peak center is shifted to lower binding energy as well as broadened either by doping and/or thermal treatment. This could be due to the incorporation of other species during the annealing process.

In addition, the deconvoluted peak around 286.8 eV tends to higher binding energy after thermal annealing. This is likely due to the formation of C-O bonds, which appear at a higher binding energy compared to their adjacent overlapped signal of the C = C bond. Moreover, the relative intensity of the C = N/C = O deconvoluted peak is decreased with doping and annealing, suggesting the removal of residual organic species and improved structural purity.

**Fig. 6:**
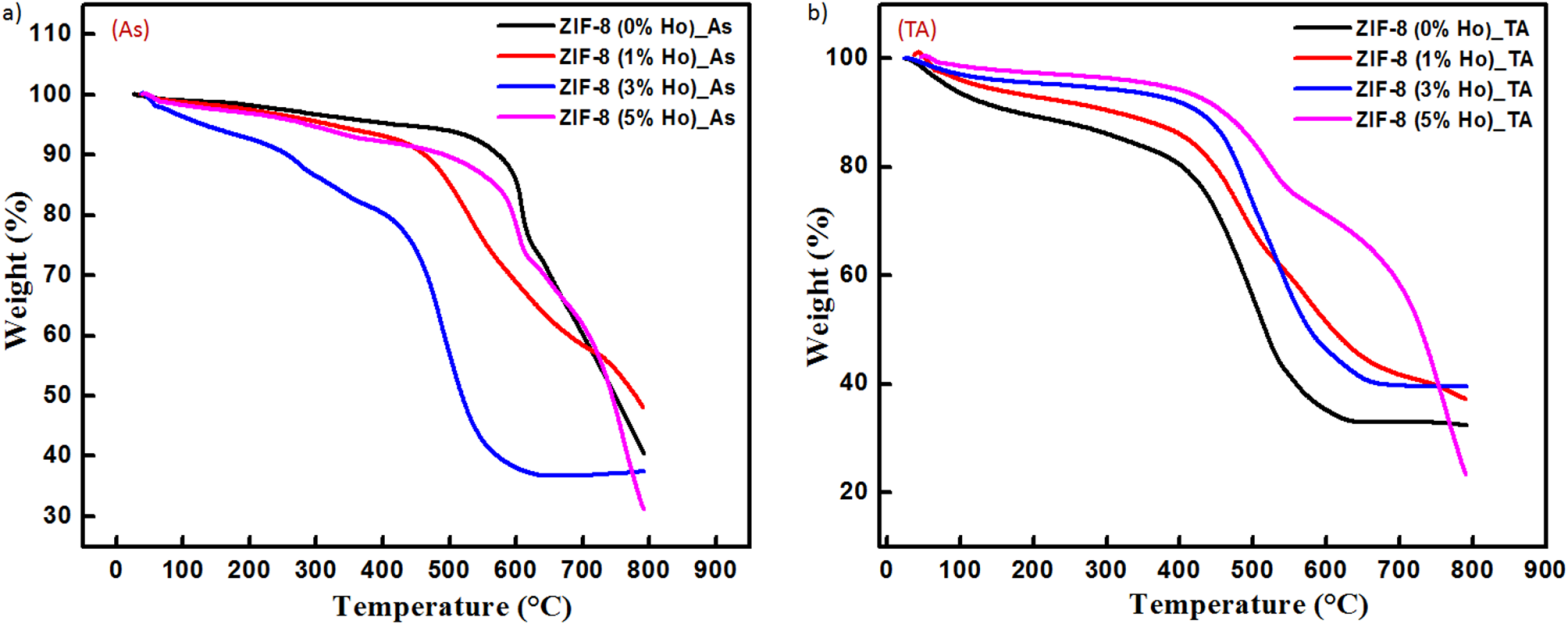
TGA curves of undoped ZIF-8 and Ho-doped ZIF-8 for (**a**) as-synthesized (As) and (**b**) thermal annealed (TA) nanocomposites.

Figure 5c presents the **Ho 4d** peak before and after annealing, which evidences the presence of Ho^3+^ in the samples. Shift to lower binding energy after annealing is observed. In addition, the band tail of the ZIF-8 (3% Ho)_As, on the left of the main peak around 163 eV, is well resolved compared to the thermal annealed one. This infers local modification in the surrounding environment around Ho^3+^ owing to the annealing process. Similar shifts to lower binding energy, due to annealing or doping, have been found in the spectra assigned to **N 1s** and **O 1s**, which could indicate a change in the local N/O coordinates with Ho^3+^. The **Zn 2p** spectra in (Fig. 5f) display two peaks, at binding energies around 1022.5 eV and 1045.6 eV, correspond to Zn 2p_3/2_ and Zn 2p_1/2_, respectively. This confirms the presence of Zn^2+^in the samples. One can also notice that the two peaks move together toward higher binding energy, by 1 eV , after doping and/or annealing, while keeping almost constant binding energy separation, about 23 eV, between them, in agreement with the reported work^35, 36^. This indicates some interactions mainly between ZIF-8 and the Ho dopant. In summary, both thermal treatment and the addition of Ho^3+^ affect the environment and chemical bonding of the ZIF-8 species. Thus, to have information about the thermal stability of the synthesized nanocomposites, thermogravimetric analyses (TGA) have been performed forZIF-8 nanocrystals as a function of Ho ion concentration, as shown in Fig. 6a, b. In Fig. 6a, the ZIF-8 (0%Ho)_As nanocrystals show that the first weight loss (1.697%) takes place in the range of 25.44–188.57 °C, which is ascribed to the desorption of surface water in the nanocrystals as well as the removal of guest molecules (methanol).The second weight loss (≈ 5.335%) occurred between 188.57 and 526.72 °C. This is caused by the release of excess of the other absorbed unreacted molecules (e.g., 2-methylimidazole) from the pore structure. Subsequently, the third and fourth stages, at 526.72–620.49 °C (≈ 17.66%) and 620.49–790.28 °C (≈ 34.91%), exhibit sharp weight loss with the increase of temperature. This indicates the decomposition and the collapse of the skeleton structure of the as-synthesized nanocomposites. Aleksandra Schejn et al.[38] reported a systematic investigation of the influence of the zinc source on the morphology and size of ZIF-8 crystals. They mentioned that the most significant feature that appeared in TGA results is the size-dependent thermal stability up to 300 °C in the case of the smaller 141 nm size nanocrystals prepared from Zn(NO_3_)_2_ and up to 390 °C in the case of the larger 1050 nm size crystals prepared from ZnBr_2_^37^. It is worth mentioning that the thermal decomposition of ZIF-8 can be affected by the dopant lanthanide ions. For instance, T.R. da Rocha et al.^38^ reported on the influence of Eu addition to the thermal decomposition of ZIF-8. They found that the coordination bonds between europium-imidazole affect the decomposition stages of the MOF, which in turn alter the thermogram curve profile. They also concluded that Eu improves the thermal stability of the compound, in contrast to our obtained result on Ho-doped ZIF. The incorporation of Ho induces alternation of thermal decomposition stages at lower temperatures compared to undoped ZIF-8. This discrepancy, between the two cases of Eu and Ho, could be due to the difference in dopant size and its reactivity for the bond formation. In addition, the effect of Ho doping on the thermal stability of ZIF-8 is illustrated as shown in Fig. 6b. It is found that the addition of Ho ions with different concentrations decreases the thermal stability of the undoped ZIF-8. However, the TGA of the heat-treated nanocomposites evidences that the doped nanocrystals reveal more resistance to the weight loss and decomposition compared to the undoped ones.

**Fig. 7:**
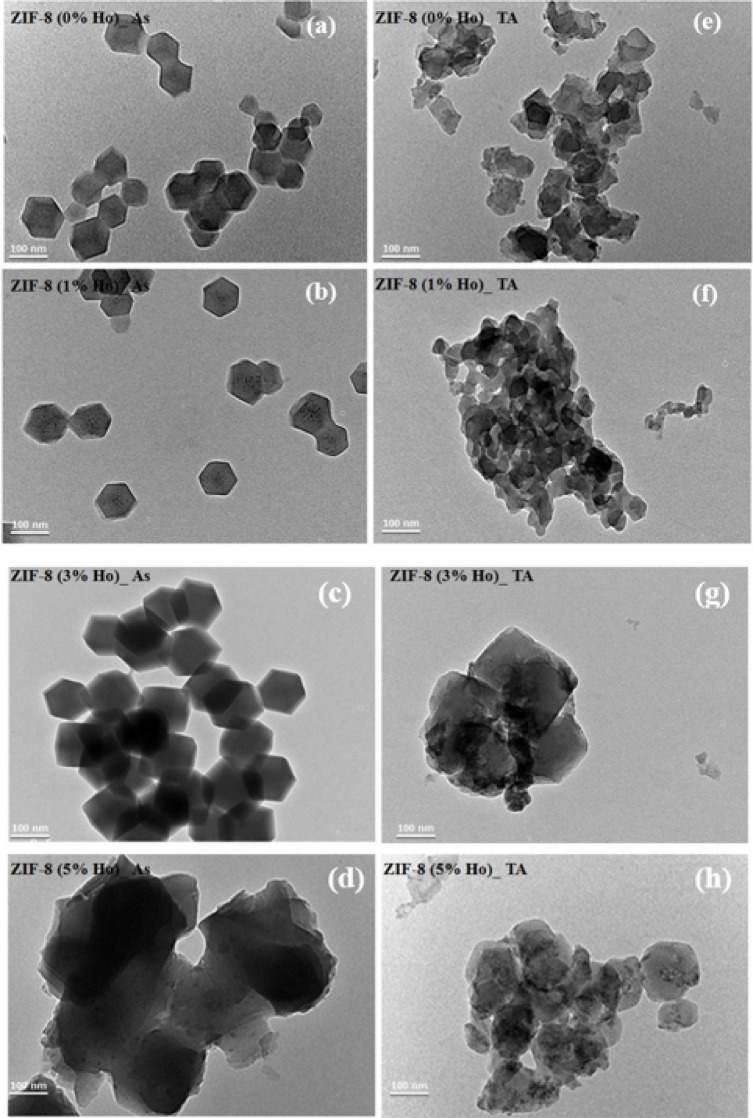
TEM images of undoped ZIF-8 and Ho-doped ZIF-8 for as-synthesized (As) and thermal annealed (TA) nanocomposites.

To figure out the local modification in the undoped ZIF-8 and Ho-doped ZIF-8 nanocomposites’ morphologies, TEM images were measured. The TEM images of undoped ZIF-8 and Ho-doped ZIF-8 NCs with different mole ratios (1–5% Ho) before and after heat treatment at 350 °C are shown in Fig. 7. ZIF-8 (0%Ho)_As displays well-dispersed nanoparticles with uniform size and distinct polyhedral morphology, consistent with the characteristic sodalite-like structure of ZIF-8 (Fig. 7a). The absence of agglomeration and the small particle size suggest highly mono-dispersed and effective framework formation^39^. Besides, ZIF-8 (1%Ho)_As NCs, as shown in Fig. 7b, the particles maintained their polyhedral shape but exhibited a slight increase in size and more defined edges, indicating successful incorporation of Ho^3+^ ions without disrupting the crystal shape or morphology. This also could imply a homogeneous distribution of dopant within the framework. Moreover, ZIF-8 (3%Ho)_As NCs, as shown in Fig. 7c, a noticeable increase in particle size is observed, accompanied by partial agglomeration. The formation of larger polyhedral crystals suggests improving the crystallinity of this sample, probably due to the enhancement of crystal growth dynamics. Further increase of the Ho mole ratio, to 5% Ho, results in significant coalescence and densification of particles as shown in Fig. 7d. The blurred boundaries and fused structures indicate that excessive doping induces structural distortion and lattice stress, potentially leading to partial collapse of the framework and loss of porosity due to dopant oversaturation. It is worth mentioning that the obtained TEM results are in agreement with XRD, which shows the increase of crystallite size with increasing the concentration of Ho^3 +^^40^.

**Fig. 8:**
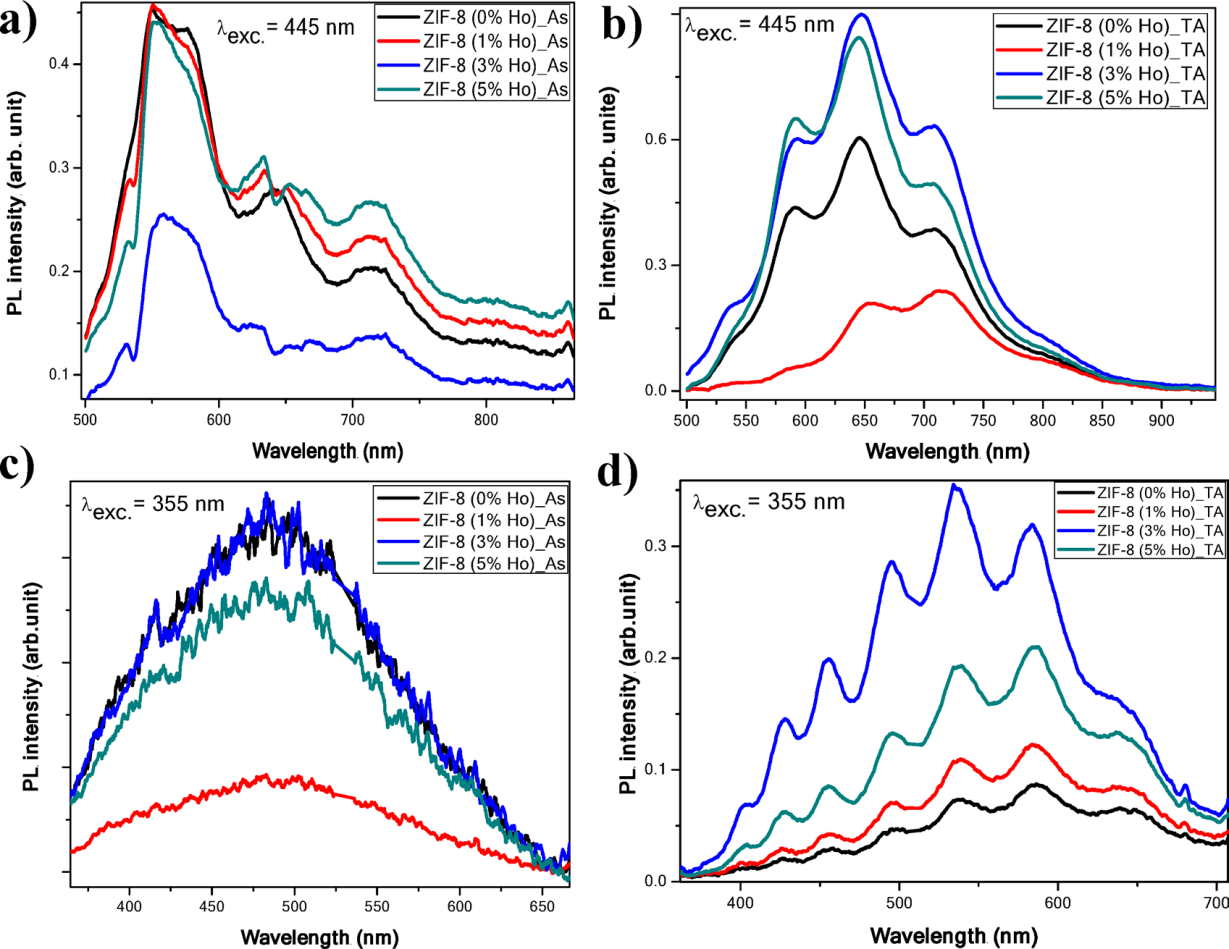
PL spectra of undoped ZIF-8 and Ho-doped ZIF-8 NCs (**a**) as-synthesized (As), (**b**) thermal annealed (TA) under excitation wavelength at 445 nm, and (**c**) as-synthesized (As), (**d**) thermal annealed (TA) under excitation wavelength at 355 nm.

On the other hand, the annealed samples exhibit a strong crystal shape deformation, especially in both cases of the ZIF-8 (0%Ho)_TA and ZIF-8 (1%Ho)_TA NCs, as shown in Fig. 7 (e, f). The other two Ho percentages (3% and 5%) reveal less structure agglomeration, with quite distinct cubic edges as shown in Fig. 7 (g, h). This is also in agreement with the XRD results, which show better crystallite diffractogram features of the ZIF-8 (3%Ho)_TA and ZIF-8 (5%Ho)_TA samples. The PL was measured for the synthesized nanocomposites before and after annealing. The PL has been performed at two excitation wavelengths using a visible laser at 445 nm and a UV laser at 355 nm. The reason behind the selection of these two excitation wavelengths is to target the excitation of the potential guest, Ho ions as dopant, and the host ZIF-8, respectively, as previously reported excitations for both^41, 42^. Figure 8 shows the collected PL spectra from all synthesized nanocomposites under the two different excitation wavelengths. It can be observed that the PL signals from the as-synthesized nanocomposites are very weak, and we had to collect them at a very long acquisition time (one order of magnitude higher than the case of PL detected from the annealed NCs).

**Fig. 9:**
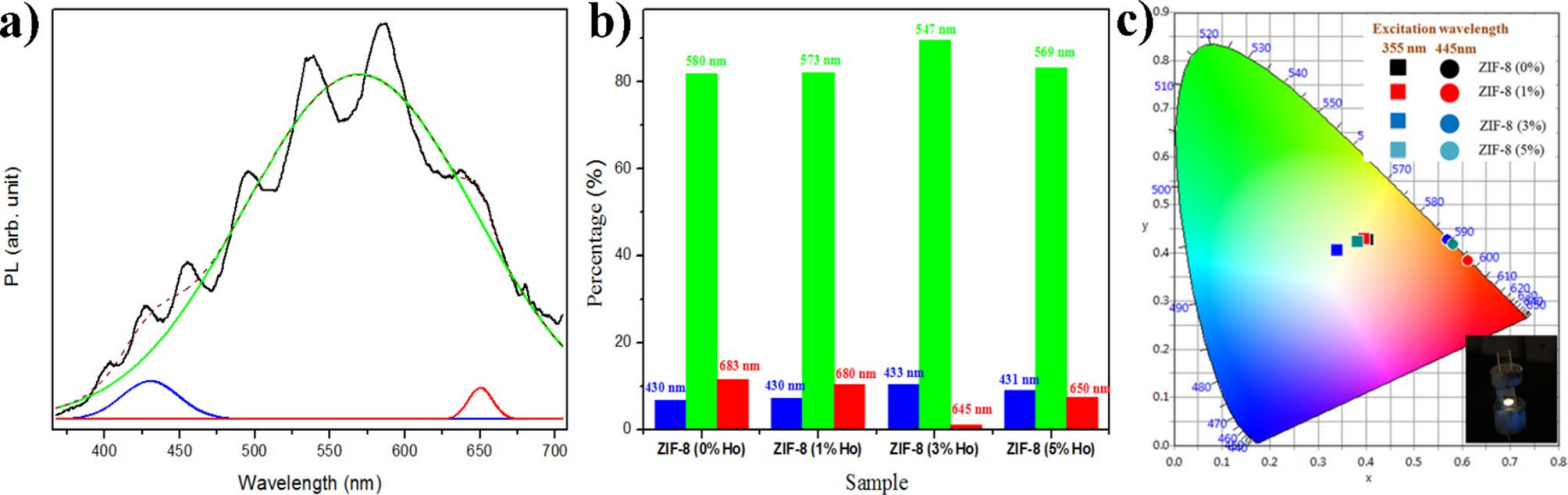
(**a**) de-convolution analysis for PL spectrum, (**b**) histogram presentation, based on the de-convolution analyses, of the contribution of each portion of colour at the corresponding colour wavelength for each sample, (**c**) CIE diagram of undoped ZIF-8 and Ho-doped ZIF-8 nanocrystals under two different excitation wavelengths at 445 nm and 355 nm for thermal annealed (TA) nanocrystals. The inset figure is a photo of the observed naked-eye white light emission.

In the case of annealed NCs, the PL signals are more intense, and the emission light can be observed by the naked eye. This indicates the significant role of the thermal treatment in order to optically activate and/or form emitting species. The PL spectra show a very broad band that covers most of the visible range. In addition, in both excitation wavelengths, the ZIF-8 (3%Ho)_TA sample reveals the highest PL intensity. Moreover, this sample exhibits an intense naked-eye observed white light emission, as depicted in Fig. 9c, under the excitation of 355 nm wavelength. However, the other samples reveal less intense white-yellowish emission. It is worth mentioning that a broad weak emission band in the blue region has been reported from ZIF-8, which is enhanced after doping with Cd ions^41^. The emission enhancement was attributed to ligand-metal charge transfer from 2-methylimidazole to Cd ions^41^. In another case, the PL of Eu-doped ZIF-8 has been reported and exhibited two spectral features. Broad emission band in the blue region and narrow emission lines, related to Eu transitions, in the red range. The broad emission band was attributed to the π* → π transition of the imidazole ligand present in the MOF^38^.Chang Liu et al. also reported on the PL response from Eu and Tb complexes doped ZIF-8 for multicolor emission purposes^33^. They found that the PL spectral features from the RE ions and the host ZIF-8 can be separately detected based on selective excitation of each species. They attributed the obtained results to the absence of energy transfer between the host and the dopant. In our study, the spectral features related to Ho ions, expected at the green region around 540 nm, couldn’t be separated due to the strong broad emission band emitted from the NCs. This implies either that the PL spectra related to Ho are overlapped by the whole emission spectrum or that the PL spectral band exclusively originates from the host. The fact that the undoped ZIF-8 emits similar PL spectrum features, although with lower intensity, indicates that the PL might be attributed to the host’s optical response. However, the increase in PL intensity with the addition of Ho points to indirect contribution of Ho to the optical process. This contribution could occur by facilitating or mediating the charge transfer between the metal and ligands, which is responsible for the emission from the host. Thanks to the resultant broad emission and to the amount of Ho, the relative amount of blue to green to red portions of the PL spectra can be modified and resulted in strong eye-observed white light emission. However, guided by literature^35^, the PL behaviour can be proposed. Considering the optical bandgap of ZIF-8 is well above 4.5 eV, the excitation by 355 nm (3.5 eV) or 445 nm (2.78 eV) is much lower than the resonant excitation from its LUMO to HOMO bands. Thus, the obtained luminescence from the host can likely be attributed to defect-related transition (Zn defects excited at 3.02 eV)^35^. Hence, this kind of radiative defects can be excited under illumination of 355 nm (3.5 eV) and could emit at 443 nm according to the proposed mechanism in^35^. Fortunately, this wavelength (443 nm) is fairly matching with the reported excitation band of Ho^3+^[32, 41].  This could imply that partial energy transfer between the host defect and Ho ions results in spectral overlapping. In this regard, spectral deconvolution has been performed using Gaussian fitting for all samples excited by 355 nm. The best deconvolution fitting shows that the PL band is composed of three sub-bands in the blue, green, and red regions, see Fig. 9a as an example. This could be due to the composition of the generated white light emission with respect to the portions of the three colours. The relative contribution of each portion of colour is estimated from the deconvolution analyses. Figure 9b depicts a histogram presentation, based on the deconvolution analyses, of the contribution of each portion of colour at the corresponding colour wavelength for each sample. It can be observed that, despite almost equal contributions of the blue (at 430 nm) portions in all samples, both peak positions and the amount of greenish-yellow relative to red portions are crucial to the quality of the white emission. This can be manifested in the position of CIE, Fig. 9c point coordination, which shows a tendency from red to greenish-yellow border with Ho concentration. The CIE, Fig. 9c, has been generated in order to investigate the colour properties emitted from the samples. It is found that the samples emit in the red CIE zone in the case of excitation by 445 nm. In the other case, under excitation by 355 nm, the CIE shows that their emission colours are close to the white zone. The change in the CIE coordination from the composite to another is due to the relative PL intensity of the spectral components in the blue, green, and red ranges.

## Conclusion

Undoped ZIF-8 and different mole ratios (1–5%) of Ho-doped ZIF-8 nanocomposites (NCs) have been prepared via chemical co-precipitation technique. The formed nanocrystals phase and structure have been examined by XRD and TEM measurements. The bond structure and thermal stability investigations indicated a mild microstructural degradation of the ZIF-8 due to the doping and heat treatment processes. However, these structural modifications induced a positive impact on the luminescence response from the nanocrystals. This resulted in a generation of white light emission when the annealed nanocomposites underwent laser excitation at 355 nm wavelength. The present study points to the significant role of the rare-earth elements doped in ZIF-8 nanophosphor for potential lighting applications.

## Data Availability

The datasets used and/or analysed during the current study available from the corresponding author on reasonable request.
